# Receptor-interacting protein kinase 3 deficiency inhibits immune cell infiltration and attenuates organ injury in sepsis

**DOI:** 10.1186/cc13970

**Published:** 2014-07-04

**Authors:** Archna Sharma, Shingo Matsuo, Weng-Lang Yang, Zhimin Wang, Ping Wang

**Affiliations:** 1 Center for Translational Research, The Feinstein Institute for Medical Research, 350 Community Dr., Manhasset, NY 11030, USA; 2 Department of Surgery, Hofstra North Shore-LIJ School of Medicine, Manhasset, NY 11030, USA; 3 Current address: Department of Surgery II, Tokyo Women’s Medical University, Tokyo, Japan

## Abstract

**Introduction:**

Sepsis is defined as a systemic hyper-inflammatory immune response, with a subsequent immune-suppressive phase, which leads to multiple organ dysfunction and late lethality. Receptor-interacting protein kinase 3 (RIPK3)-dependent necrosis is implicated in driving tumor necrosis factor alpha (TNF-α)- and sepsis-induced mortality in mice. However, it is unknown if RIPK3 deficiency has any impact on immune cell trafficking, which contributes to organ damage in sepsis.

**Methods:**

To study this, male wild-type (WT) and RIPK3-deficient (*Ripk3*^-/-^) mice on C57BL/6 background were subjected to sham operation or cecal ligation and puncture (CLP)-induced sepsis. Blood and tissue samples were collected 20 hours post-CLP for various measurements.

**Results:**

In our severe sepsis model, the mean survival time of *Ripk3*^-/-^ mice was significantly extended to 68 hours compared to 41 hours for WT mice. *Ripk3*^-/-^ mice had significantly decreased plasma levels of TNF-α and IL-6 and organ injury markers compared to WT mice post-CLP. In the lungs, *Ripk3*^-/-^ mice preserved better integrity of microscopic structure with reduced apoptosis, and decreased levels of IL-6, macrophage inflammatory protein (MIP)-2 and keratinocyte-derived chemokine (KC), compared to WT. In the liver, the levels of MIP-1, MIP-2 and KC were also decreased in septic *Ripk3*^-/-^ mice. Particularly, the total number of neutrophils in the lungs and liver of *Ripk3*^-/-^ mice decreased by 59.9% and 66.7%, respectively, compared to WT mice post-CLP. In addition, the number of natural killer (NK) and CD8T cells in the liver decreased by 64.8% and 53.4%, respectively, in *Ripk3*^-/-^ mice compared to WT mice post-sepsis.

**Conclusions:**

Our data suggest that RIPK3 deficiency modestly protected from CLP-induced severe sepsis and altered the immune cell trafficking in an organ-specific manner attenuating organ injury. Thus, RIPK3 acts as a detrimental factor in contributing to the organ deterioration in sepsis.

## Introduction

Sepsis afflicts >800,000 people annually with the mortality rate as high as 30% [1] which accounts for up to 39% of total hospital costs in the United States [2], yet there is a lack of effective therapies for the sickest patients in intensive care units [3]. Sepsis is an acute clinical condition defined as an initial systemic hyper-inflammatory immune response associated with a proven or suspected infection, which eventually progresses to multiple organ dysfunction (severe sepsis), intractable hypotension (septic shock) and lethality [4]. Along with inflammation designed to eliminate the underlying pathogen, immune-suppressive mechanisms are also initiated during sepsis which result in immune dysfunctions and secondary infections [5,6]. An exaggerated and sustained proinflammatory cytokine response to the pathogen-associated molecular patterns (PAMPs) from invading microorganisms and damage-associated molecular patterns (DAMPs) released from dying cells in the circulation, as well as an excessive organ infiltration of leukocytes resulting in organ failure, are hallmarks of severe sepsis [7,8]. Sepsis is also characterized by the massive cell death of various cell types including immune cells such as lymphocytes and non-immune cells, such as intestinal cells and hepatocytes [9-11]. Cell death occurs through morphologically distinct processes of apoptosis and necrosis. Unlike apoptosis which is programmed cell death, necrosis has been considered as an accidental and unregulated form of cell death occurring due to metabolic failure [12,13]. Until recently, it has been recognized that the process of necrotic cell death induced by TNF-α can be programmed as apoptosis, hence called necroptosis [14,15].

The receptor-interacting protein kinase 3 (RIPK3) is a member of the family of serine/threonine protein kinases with a unique C-terminal domain, while its N-terminal kinase domain is similar to other RIPK family members including RIPK1, RIPK2 and RIPK4 [16-18]. RIPK3 was shown to function as a molecular switch between TNF-α-induced apoptosis and necrosis via interaction with RIPK1 forming a necrosis signaling complex (necrosome) for triggering necroptosis [19-23]. Recently, RIPK3-dependent necrosis has been shown to be responsible for the mortality of septic mice induced either by TNF-α injection or polymicrobial sepsis, suggesting that targeting necrotic cells could be a novel and promising therapeutic approach for improving sepsis outcome [24,25]. RIPK3-mediated necroptosis can initiate a ‘cell death storm’ in sepsis with the release of DAMPs, such as high-mobility group box 1 (HMGB1), IL-1α, DNA fragments, mitochondrial content, uric acid, ATP, heat shock proteins and cold-inducible RNA-binding protein (CIRP) to provoke a strong inflammatory response [24,26,27]. However, the association of RIPK3 with leukocyte recruitment under such conditions remains to be studied. Hence, determining the effects of ablation of RIPK3-mediated necrosis on the trafficking of various subtypes of immune cells and resultant organ injury following sepsis is critical for the development of therapies for patients.

In the present study, using RIPK3-deficient (*Ripk3*^*-/-*^) mice in a physiologically relevant model of severe polymicrobial sepsis induced by cecal ligation and puncture (CLP), we examined the role of RIPK3 in altering immune cell trafficking and organ injury in sepsis. We showed that RIPK3 deficiency significantly prolonged the survival and attenuated acute lung and liver injury in severe septic mice. Furthermore, RIPK3 deficiency resulted in the reduction of infiltration of neutrophils, natural killer (NK) cells and CD8^+^ T lymphocytes in an organ specific manner. Thus, RIPK3 played a crucial role in altering immune cell trafficking during sepsis, which may suggest targeting RIPK3 as a therapeutic strategy in attenuating organ injury caused by excessive immune cell infiltration in sepsis.

## Material and methods

### Mice

C57BL/6 mice matched for gender and age were either purchased from Taconic (Albany, NY, USA) or bred in our own facility and used as wild type (WT) control mice. *Ripk3*^*-/-*^ mice [28] were provided by V. Dixit (Genentech, San Francisco, CA, USA). Male C57BL/6 or *Ripk3*^*-/-*^ mice were used at the age of 8- to 12-weeks. Mice were housed in a temperature-controlled room on a 12 hour light/dark cycle and fed a standard laboratory diet within the Feinstein Institute for Medical Research (Manhasset, NY, USA). All experiments were performed in accordance with the guidelines for the use of experimental animals by the National Institutes of Health (Bethesda, MD, USA) and were approved by the Institutional Animal Care and Use Committee (IACUC) at the Feinstein Institute for Medical Research.

### Cecal ligation and puncture

CLP was used as a model of polymicrobial sepsis. The mice were anesthetized by isoflurane inhalation, and the abdomen was shaved and washed with 10% povidone iodine. A 1- to 2-cm midline incision was performed to allow exposure of the cecum and tightly ligated 1.5 cm from the tip with a 4–0 silk suture. Double puncture of the cecum with a 22-gauge needle was performed. The cecum was then gently squeezed to extrude sufficient amount of feces from the perforation sites and returned to the peritoneal cavity. The laparotomy site was then closed with 6–0 silk suture. Sham operated animals underwent the same procedure with the exception that the cecum was neither ligated nor punctured. The CLP animals were resuscitated with 1 ml of isotonic sodium chloride solution by subcutaneous injection immediately after the surgery to improve dehydration after CLP. At 20 hours after CLP or sham operation, mice were anesthetized and blood, liver and lungs were collected. A section of lung tissue was preserved in formalin for histopathology. Blood samples were centrifuged at 3,000 *g* for 10 minutes to collect plasma. The plasma and the remainder of the tissue samples were frozen immediately in liquid nitrogen and stored at -80°C until analysis. An additional set of experiments was performed for harvesting the liver and lungs for leukocyte preparation 20 hours post-CLP or sham. For the survival study, mice were administered the antibiotic, Primaxin (Merck & Co., Whitehouse Station, NJ, USA) (0.5 mg/kg) subcutaneously following the CLP procedure and were monitored for seven days to record survival.

### Measurements of pro-inflammatory cytokines and organ injury markers

Pro-inflammatory cytokines TNF-α and IL-6 were quantified by using the specific mouse ELISA kits (BD Biosciences, Franklin Lakes, NJ, USA) in plasma and lung tissue. Plasma levels of aspartate aminotransferase (AST), alanine aminotransferase (ALT) and lactate dehydrogenase (LDH) were measured using commercial assay kits (Pointe Scientific, Lincoln Park, MI, USA) according to the manufacturer’s instructions.

### Histologic examination and assessment of organ injury

The lung tissue was fixed in 10% formalin and later embedded in paraffin. The tissue blocks were then cut into 5 μm sections, transferred to glass slides and stained with H & E. Morphologic alterations in the lung tissues were examined by light microscopy and documented by photographs. Lung injury was assessed using a blinded, semiquantitative scoring system as absent, mild, moderate, or severe injury (score 0 to 3, respectively) according to the following pathological features: (1) focal alveolar membrane thickening, (2) capillary congestion, (3) intra-alveolar hemorrhage, (4) interstitial leukocyte infiltration, and (5) intra-alveolar leukocyte infiltration, and a cumulative total histology score was determined.

### Leukocyte suspension preparation from liver and lungs

Hepatic leukocytes were isolated from livers that were minced and homogenized in PBS with 1% fetal bovine serum (FBS), followed by density gradient centrifugation (2,000 rpm, 20 minutes) using 44% and 66% Percoll (GE Healthcare Bio-Sciences AB, Uppsala, Sweden). Lung leukocytes were isolated by digesting minced lungs in Roswell Park Memorial Institute (RPMI) medium containing 100 U/ml collagenase type 1 (Worthington Biochemical, Lakewood, NJ, USA) and 20 U/ml DNase 1 (Roche Diagnostics, Mannheim, Germany) for 30 minutes at 37°C in a shaker incubator followed by Percoll-gradient centrifugation. Cells at the interface and below were collected, washed and counted.

### Antibodies and flow cytometry

Single cell suspensions (1 × 10^6^ cells) obtained from different organs were pre-incubated with anti–CD16/CD32 (93) to block FcγRII/III receptors and stained with the desired antibodies (Abs) on ice for 30 minutes and analyzed on FACSVerse (BD Bioscience, San Jose, CA, USA). The following anti-mouse Abs conjugated to fluorescein isothiocyanate (FITC), phycoerythrin (PE), peridinin chlorophyll protein-cyanine 5.5, allophycocyanin or PE-cyanine7 (all from Biolegend, San Diego, CA, USA) were used for staining: anti-Gr-1 (RB6-8C5) and anti-CD11b (M1/70), anti-CD4 (GK1.5/RM4-5), anti-CD8α (53–6.7), anti-TCRβ (H57-597), anti-NK1.1 (PK136). Dead cells were excluded by forward light scatter or forward light scatter plus propidium iodide. All the data were acquired and presented on log scale. Data were analyzed by FlowJo software (Tree Star, Ashland, OR, USA).

### Myeloperoxidase activity assay

Lung tissues were homogenized in potassium phosphate buffer containing 0.5% hexa-decyl-trimethyl-ammonium bromide. After centrifugation the supernatant was diluted in reaction solution, and the rate of change in optical density (OD) for two minutes was measured at 460 nm to calculate myeloperoxidase (MPO) activity.

### Real-time RT-PCR analysis

Total RNA was extracted from lung and liver tissues using TRIzol reagent (Invitrogen, Carlsbad, CA, USA) and was reverse-transcribed into cDNA using murine leukemia virus reverse transcriptase (Applied Biosystems, Foster City, CA, USA). A PCR reaction was carried out in 25 μl of a final volume containing 0.1 μM of each forward and reverse primer, cDNA and 12.5 μl SYBR Green PCR Master Mix (Life Technologies, Grand Island, NY, USA). Amplification was conducted using an Applied Biosystems 7300 real-time PCR machine (Applied Biosystems) and analyzed by the 2^-ddCt^ method for relative quantization normalized to mouse β-actin mRNA. Relative expression of mRNA was expressed as the fold change in comparison with the sham tissues. The primers used for this study are listed in Table 1.

**Table 1:** Sequences of the primers used in RT-PCR

**Gene name**	**Forward primer sequence**	**Reverse primer sequence**
*Cxcl3* (MIP-1)	AGGTCCCTGTCATGCTTCTG	TCTGGACCCATTCCTTCTTG
*Cxcl2* (MIP-2)	CCCTGGTTCAGAAAATCATCCA	GCTCCTCCTTTCCAGGTCAGT
*Cxcl1* (KC)	GCTGGGATTCACCTCAAGAA	ACAGGTGCCATCAGAGCAGT
*Actb* (β-actin)	CGTGAAAAGATGACCCAGATCA	TGGTACGACCAGAGGCATACAG

### TUNEL assay

The presence of apoptotic cells in the lung sections were assessed using a TUNEL staining kit (Roche Diagnostics) according to the manufacturer’s instructions. The negative control was performed by incubating slides in the mixture containing only deoxynucleotidyl transferase. TUNEL-positive cells were counted in five fields/section under a fluorescence microscope (×200), and the number of cells/field are shown.

### Western blotting

Lung tissue was homogenized by sonication in lysis buffer (10 mM Tris–HCl, pH 7.5, 120 mM NaCl, 1% NP-40, 1% sodium deoxycholate and 0.1% SDS) containing a protease inhibitor cocktail (Roche Diagnostics). Total lung lysates were fractionated on Bis-Tris gels (4% to 12%), transferred to nitrocellulose membrane, immunoblotted with anti-cleaved caspase-3 or anti-cleaved caspase-8 (Cell Signaling Technology, Beverly, MA, USA) or anti-β-actin (Sigma-Aldrich, St Louis, MO, USA) Abs and visualized by using Pierce ECL 2 Western Blotting Substrate (Thermo Scientific, Southfield, MI, USA).

### Statistical analysis

Data are expressed as mean ± standard error of the mean (SEM) and analyzed using SigmaPlot11 graphing and statistical analysis software (Systat Software Inc., San Jose, CA, USA). Multiple groups were compared by one-way analysis of variance (ANOVA) using the Student-Newman-Keuls’ (SNK) test. Student’s *t* test was used for two-group analysis. The survival data were analyzed using the Kaplan-Meier method and compared by the log-rank test. Differences in values were considered significant if *P* <0.05.

## Results

### RIPK3 deficiency prolongs survival and reduces systemic injury in CLP-induced acute sepsis

**Figure 1:**
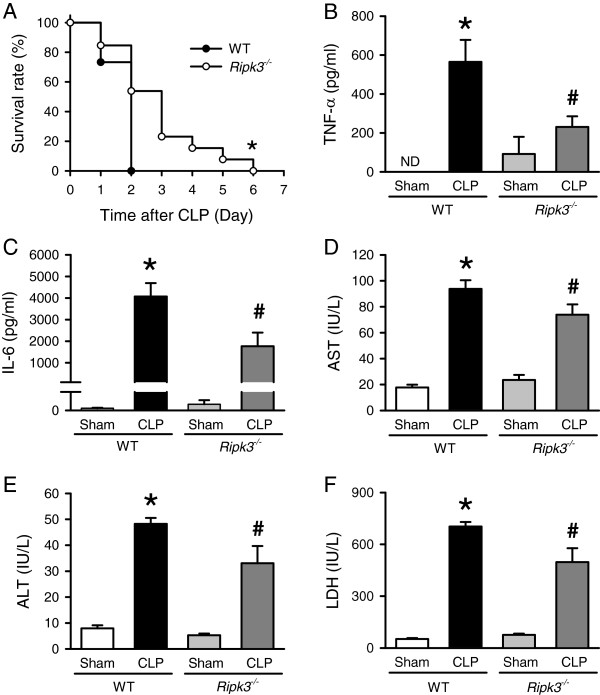
***Ripk3***^***-/- ***^**mice have prolonged survival and reduced systemic injury after CLP. (A)** WT (n = 15) and *Ripk3*^*-/-*^ mice (n = 13) were subjected to CLP as described in Material and Methods for a seven-day survival study. *P <0.05 versus WT. **(B-F)** WT and *Ripk3*^*-/-*^ mice underwent sham- or CLP-operation and blood samples were collected at 20 hours after CLP for the analysis of plasma levels of the cytokines TNF-α **(B)** and IL-6 **(C)** and serum organ injury markers AST **(D)**, ALT **(E)** and LDH **(F)**. Data are expressed as mean ± SEM (n = 5 per group). *P <0.05 versus WT sham, ^#^*P* <0.05 versus WT CLP. ND: nondetectable. ALT, alanine aminotransferase; AST, aspartate aminotransferase; CLP, cecal ligation and puncture; SEM, standard error of the mean; WT, wild type.

We first performed a seven-day survival study on WT and *Ripk3*^*-/-*^ mice to confirm the effect of RIPK3 deficiency on a very severe model of CLP-induced polymicrobial sepsis. The WT mice group had 100% mortality on day 2, whereas *Ripk3*^*-/-*^ mice group had 100% mortality on day 6 (Figure 1A). The mean survival time of septic WT mice was about 41 hours, whereas that of septic *Ripk3*^*-/-*^ mice was about 68 hours. Therefore, RIPK3 deficiency exerted a protective effect as shown by prolonging survival of severely septic mice.

Since excessive elevation of pro-inflammatory cytokines in circulation is a major contributor to remote organ injury after CLP, we then examined the plasma levels of pro-inflammatory cytokines. TNF-α and IL-6 plasma levels were both significantly increased in WT and *Ripk3*^*-/-*^ mice 20 hours after CLP, compared to each sham group (Figure 1B, 1C). However, the levels of TNF-α and IL-6 in septic *Ripk3*^*-/-*^ mice were 59.2% and 56.6%, respectively, lower than those in septic WT mice (Figure 1B, 1C). Similarly, the serum levels of the organ injury markers AST, ALT, and LDH were all significantly increased in WT and *Ripk3*^*-/-*^ mice 20 hours after CLP, compared to each sham group (Figure 1D-F). The levels of AST, ALT and LDH in septic *Ripk3*^*-/-*^ mice were 21.2%, 31.5% and 29.2%, respectively, lower than those in septic WT mice (Figure 1D-F). Taken together, these results indicated that RIPK3 deficiency resulted in the reduction of pro-inflammatory cytokines and organ injury markers in septic mice.

### RIPK3 deficiency attenuates CLP-induced damage of lungs

**Figure 2:**
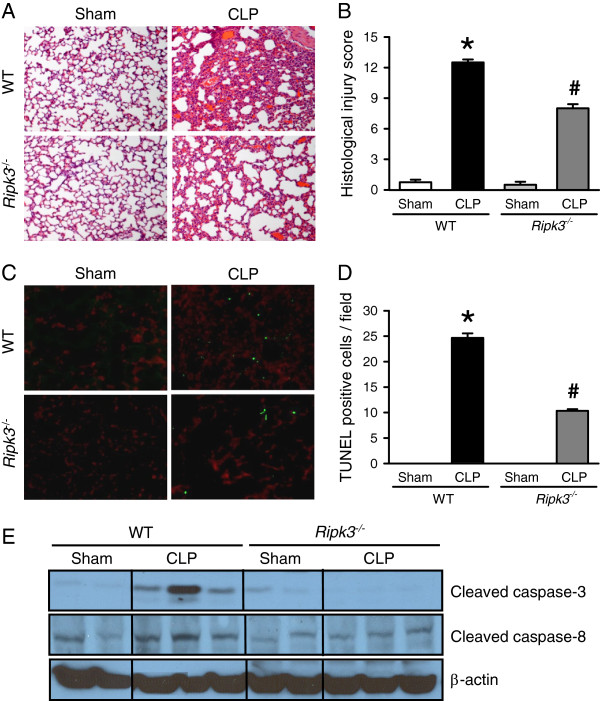
***Ripk3***^***-/- ***^**mice have attenuated lung damage after CLP.** The lung tissues from WT and *Ripk3*^*-/-*^ mice were harvested 20 hours after sham- or CLP-operation. **(A)** Representative lung histology sections (H & E staining; original magnification × 200) and **(B)** the lung injury scores from the above mentioned mice groups determined as described in Materials and Methods are shown. **(C)** Representative TUNEL staining (green fluorescent) and nuclear counterstaining (red fluorescent) of lung sections (original magnification × 200) and **(D)** the number of apoptotic cells quantified from TUNEL staining (averaged over 10 microscopic fields per mouse) is shown. **(E)** Representative blots for cleaved caspase-3 and caspase-8 in the lung tissues are presented and β-actin is used as a loading control. Data expressed as means ± SEM (n = 5 per group). *P <0.05 versus WT sham and ^#^*P* <0.05 versus WT CLP. CLP, cecal ligation and puncture; SEM, standard error of the mean; WT, wild type.

CLP-induced sepsis resulted in acute lung injury (ALI) and lung dysfunction is the first step in the development of multiple organ failure. The lung tissues of WT mice 20 hours after CLP showed substantial morphological changes, including alveolar collapse, edema, hemorrhage and infiltration of inflammatory cells (Figure 2A, upper). In contrast, the lung tissues of septic *Ripk3*^*-/-*^ mice exhibited less microscopic deterioration (Figure 2A, lower). As quantified in Figure 2B, the lung histological injury score in septic WT mice significantly increased by 16.7 fold compared to the sham group, while this score was reduced by 36% in septic *Ripk3*^*-/-*^ mice. These data suggest that RIPK3 deficiency improved lung morphology in CLP-induced severe sepsis.

To investigate the effect of RIPK3 deficiency on apoptosis, a TUNEL assay, which is a common method for detecting DNA fragmentation, was conducted by immunohistochemistry in the lung tissues. The TUNEL-positive cells in the lung tissues of WT and *Ripk3*^*-/-*^ mice were well detected after CLP compared to the sham groups (Figure 2C). However, the number of TUNEL-positive cells in the lung tissues of *Ripk3*^*-/-*^ mice after CLP was significantly reduced by 58.1% compared with WT mice after CLP (Figure 2D). In addition, expression of cleaved caspase-3 and caspase-8 in the lung tissues of WT mice was increased after CLP compared to the sham procedure, whereas such an increase was not observed in *Ripk3*^*-/-*^ mice after CLP (Figure 2E). Our data suggest that absence of RIPK3 also protects lungs from apoptotic cell death in ALI induced by sepsis.

### RIPK3 deficiency reduces neutrophil infiltration in the lungs after CLP

**Figure 3:**
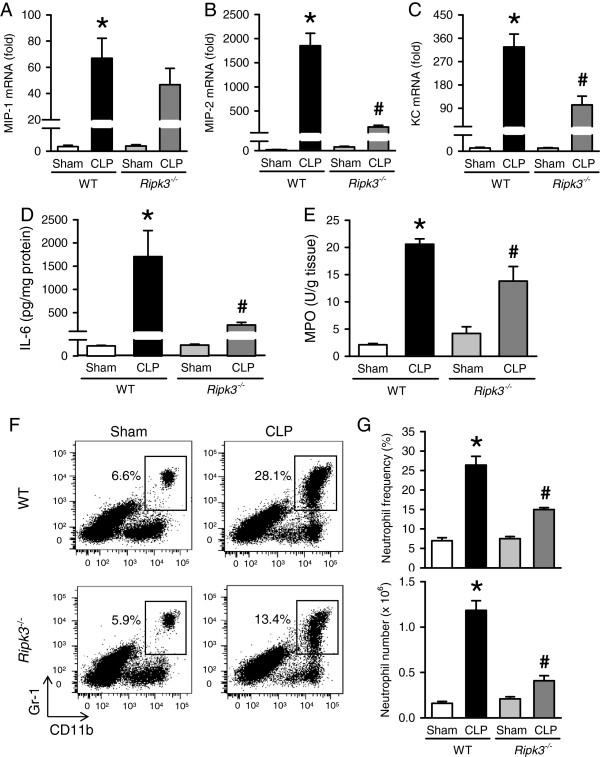
***Ripk3***^***-/- ***^**mice show decreased chemokine expression and neutrophil infiltration in the lungs after CLP.** The lung tissues from WT and *Ripk3*^*-/-*^ mice were harvested 20 hours after sham- or CLP- operation. **(A-C)** The mRNA levels of MIP-1 **(A)**, MIP-2 **(B)** and KC **(C)** in the lungs were determined by real-time RT-PCR analysis and expressed as fold change relative to WT sham. **(D)** Lung levels of IL-6 were determined by ELISA. **(E)** Lung myeloperoxidase (MPO) activity was determined spectrophotometrically. **(F)** Flow cytometric analysis of surface Gr-1/CD11b expression on gated live lung leukocytes from sham- or CLP-operated WT and *Ripk3*^*-/-*^ mice. Numbers adjacent to outlined areas show the percentage of Gr-1^+^CD11b^+^ neutrophils in the representative dot blots as indicated. **(G)** The graphs show percentage (upper) and absolute numbers (lower) of Gr-1^+^CD11b^+^ neutrophils in the lungs as indicated. Data are expressed as mean ± SEM (n = 5 per group). *P <0.05 versus WT sham and ^#^*P* <0.05 versus WT CLP. CLP, cecal ligation and puncture; KC, keratinocyte-derived chemokine; MIP, macrophage inflammatory protein; SEM, standard error of the mean; WT, wild type.

ALI also results in local proinflammatory cytokine and chemokine overproduction, so we checked their expression in the lungs. The mRNA levels of macrophage inflammatory protein 1 (MIP-1), MIP-2, and keratinocyte-derived chemokine (KC) determined by RT-PCR increased by 67-, 1,852-, and 327-fold, respectively, in WT mice after CLP (Figure 3A-C). Although the mRNA levels of MIP-1, MIP-2, and KC were also increased in septic *Ripk3*^*-/-*^ mice, their levels were reduced by 30.2%, 90.9%, and 68.4%, respectively, compared to the septic WT mice (Figure 3A-C). Similarly, IL-6 protein levels in the lungs of septic *Ripk3*^*-/-*^ mice were decreased by 86.2% compared to those in septic WT mice (Figure 3D).

Chemokines mediate neutrophil infiltration into the lungs, which is the hallmark of ALI. We determined the MPO activity which reflects neutrophil infiltration. As expected, lung MPO activity of septic WT mice was significantly increased by 9.8-fold compared to sham (Figure 3E). Septic *Ripk3*^*-/-*^ mice showed significantly reduced (down by 32.9%) lung MPO activity as compared with that of septic WT mice. To further confirm the neutrophil infiltration into the lungs, we processed the lung tissues to isolate leukocytes and then stained them with Gr-1 and CD11b surface markers to identify neutrophils. Frequency and the number of Gr-1^+^CD11b^+^ neutrophils in the lungs of WT mice was significantly increased by 3.8- and 7.1-fold, respectively, after CLP as compared to sham WT mice (Figure 3F, 3G). Such increase in frequency and the number of neutrophils was reduced by 43.3% and 59.9%, respectively, in septic *Ripk3*^*-/-*^ mice in comparison with septic WT mice (Figure 3F, 3G). These results collectively show that RIPK3 deficiency attenuates lung neutrophil infiltration associated with reduction of chemokines and cytokines.

### RIPK3 deficiency attenuates neutrophil infiltration in liver after CLP

**Figure 4:**
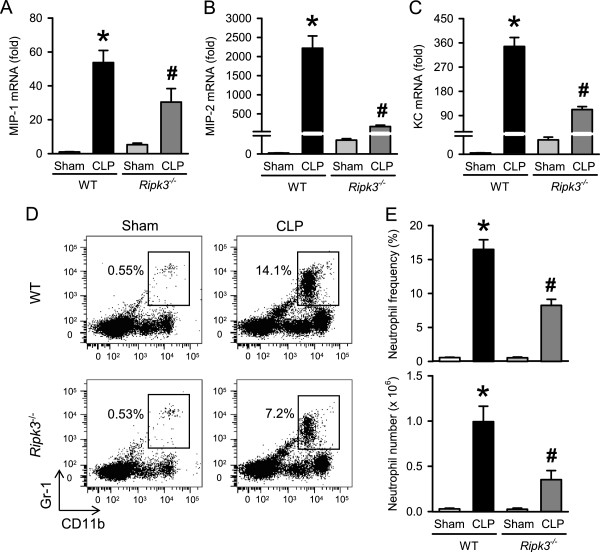
***Ripk3***^***-/- ***^**mice show decreased chemokine expression and neutrophil infiltration in liver after CLP.** The liver tissues from WT and *Ripk3*^*-/-*^ mice were harvested 20 hours after sham- or CLP-operation. **(A-C)** The mRNA levels of MIP-1 **(A)**, MIP-2 **(B)**, and KC **(C)** in the liver tissues were determined by real-time RT-PCR analysis and expressed as fold change relative to WT sham. **(D)** Flow cytometric analysis of surface Gr-1/CD11b expression on gated live hepatic leukocytes from sham- or CLP-operated WT and *Ripk3*^*-/-*^ mice. Numbers adjacent to outlined areas show the percentage of Gr-1^+^CD11b^+^ neutrophils in the representative dot blots as indicated. **(E)** The graphs show percentage (upper) and absolute numbers (lower) of Gr-1^+^CD11b^+^ neutrophils in the livers as indicated. Data are expressed as mean ± SEM (n = 5 per group). *P <0.05 versus WT sham and ^#^*P* <0.05 versus WT CLP. CLP, cecal ligation and puncture; KC, keratinocyte-derived chemokine; MIP, macrophage inflammatory protein; SEM, standard error of the mean; WT, wild type.

Since ALT is a relatively specific indicator of liver damage and it was found to be increased in septic mice, we then examined the neutrophil infiltration in acute liver injury induced by sepsis. First, we determined the mRNA levels of MIP-1, MIP-2 and KC by RT-PCR and showed a 54-, 2,218- and 346-fold increase, respectively, in septic WT mice, compared to sham WT mice (Figure 4A-C). The mRNA levels of MIP-1, MIP-2 and KC in septic *Ripk3*^*-/-*^ mice were reduced by 43.3%, 92.4% and 67.5%, respectively, as compared with the septic WT mice (Figure 4A-C). The frequency and number of Gr1^+^CD11b^+^ neutrophils in the liver of WT mice were significantly increased by 27.2- and 43.6-fold, respectively, after CLP as compared to the sham WT mice (Figure 4D, 4E). Such increase in the frequency and the number of neutrophil was reduced by 51.7% and 66.7%, respectively, in septic *Ripk3*^*-/-*^ mice in comparison with septic WT mice (Figure 4D, 4E). Together, the data suggest that similar to lung injury, RIPK3 deficiency also attenuates the CLP-induced liver injury partly by reducing the chemokine-mediated neutrophil infiltration.

### RIPK3 deficiency inhibits the elevation of natural killer and CD8T cells in liver after CLP

**Figure 5:**
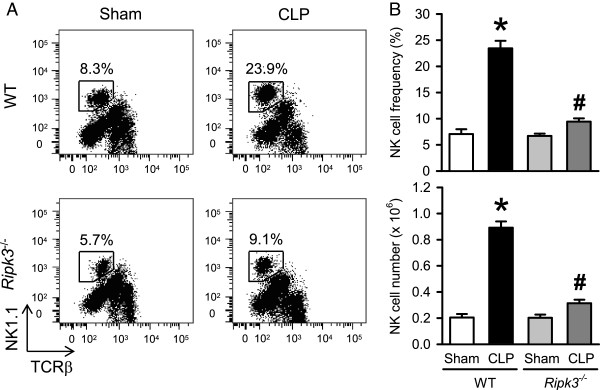
***Ripk3***^***-/- ***^**mice show decreased NK infiltration in liver after CLP.** The liver tissues from WT and *Ripk3*^*-/-*^ mice were harvested 20 hours after sham- or CLP-operation and hepatic leukocytes were isolated. **(A)** Flow cytometric analysis of surface NK1.1/TCRβ expression on gated live hepatic leukocytes. Numbers adjacent to outlined areas show the percentage of NK cells (NK1.1^+^TCRβ^-^) in the representative dot blots as indicated. **(B)** The graphs show the percentage and absolute numbers of NK cells in the liver as indicated. Data are expressed as mean ± SEM (n = 8 per group). *P <0.05 versus WT sham and ^#^*P* <0.05 versus WT CLP. CLP, cecal ligation and puncture; NK, natural killer; SEM, standard error of the mean; WT, wild type.

Apart from neutrophils, migrating natural killer (NK) cells are also known to facilitate inflammation and injury in sepsis [29]. So we examined the NK cells in the liver of WT and *Ripk3*^*-/-*^ mice under sham and CLP conditions 20 hours post-operation. To evaluate the NK cells, we stained hepatic leukocytes with NK1.1 and TCRβ surface markers to define NK1.1^+^TCRβ^-^ NK cells. The frequency and number of NK cells in the liver of WT mice were significantly increased after CLP by 3.8- and 4.4-fold, respectively, as compared to sham WT mice (Figure 5A, 5B). Whereas, the frequency and number of NK cells in septic *Ripk3*^*-/-*^ mice were decreased by 61.7% and 64.8%, respectively, in comparison with those in septic WT mice (Figure 5A, 5B).

**Figure 6:**
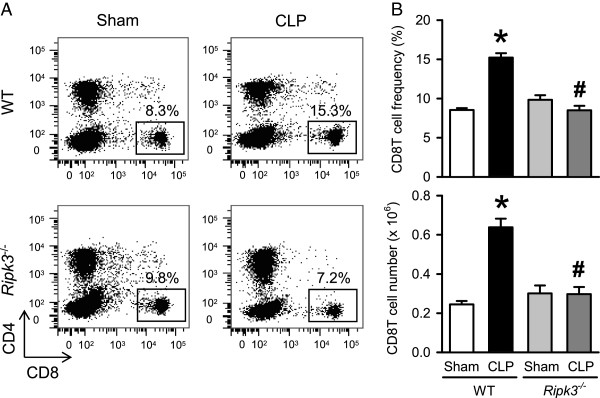
***Ripk3***^***-/- ***^**mice show decreased CD8T cells in liver after CLP.** The liver tissues from WT and *Ripk3*^*-/-*^ mice were harvested 20 hours after sham- or CLP-operation and hepatic leukocytes were isolated. **(A)** Flow cytometric analysis of surface CD4/CD8 expression on gated live hepatic lymphocytes. Numbers adjacent to outlined areas show the percentage of CD8T cells in the representative dot blots as indicated. **(B)** The graphs show the percentage and absolute numbers of CD8T cells in the livers as indicated. Data are expressed as mean ± SEM (n = 8 per group). *P <0.05 versus WT sham and ^#^*P* <0.05 versus WT CLP. CLP, cecal ligation and puncture; SEM, standard error of the mean; WT, wild type.

Combined deficiency of CD8T and NK cells is known to be beneficial in CLP-induced murine sepsis [30,31]. Also, CLP-induced sepsis is known to result in reduced numbers of peripheral CD8T cells. In contrast, a marked increase in hepatic CD8T cells which induces FasL-mediated inflammation and hepatocyte apoptosis has been reported [32]. So we examined the CD8T in liver of WT and *Ripk3*^*-/-*^ mice under sham and CLP conditions 20 hours post-operation. To evaluate the lymphocytes, we stained hepatic leukocytes with CD4 and CD8 surface markers to identify CD4T and CD8T cells. The frequency and number of CD8T cells in the liver of WT mice were significantly increased after CLP by 1.8- and 2.6-fold, respectively, as compared to sham WT mice 022 (Figure 6A, 6B) whereas, the frequency and number of CD8T cells in septic *Ripk3*^*-/-*^ mice were decreased by 44.2% and 53.4%, respectively, in comparison with those in septic WT mice (Figure 6A, 6B). These data collectively suggest that RIPK3 mediates liver specific NK and CD8T cell trafficking in CLP-induced sepsis possibly contributing to liver injury.

## Discussion

In this study, we demonstrate that RIPK3 deficiency prolongs survival in severe septic mice induced by CLP and reduces systemic levels of pro-inflammatory cytokines as well as organ injury markers. In particular, we show that RIPK3 deficiency attenuates CLP-induced ALI resulting in improved integrity of lung architecture, reduced apoptotic cell death in lung epithelium, and decreased local pro-inflammatory cytokine and chemokine production. Finally, we demonstrate that RIPK3 deficiency reduces CLP-induced neutrophil infiltration in the lungs, whereas in the liver it not only decreases the neutrophil infiltration but also decreases NK and CD8T cell accumulation. In the light of these observations we propose that RIPK3 deficiency attenuates CLP-induced organ injury by inhibiting the infiltration of immune cells in an organ-specific manner and, thus, protects against sepsis. Whether the alteration in immune cell trafficking is a direct result of RIPK3 deficiency or an indirect response to reduced necrotic DAMPs remains to be established.

Sepsis-induced cell death has been observed in various organs and cell types in murine models. For example, an increase of apoptosis has been reported in the lungs, spleen, thymus, gut epithelial cells and gut-associated lymphoid tissues [6,33,34]. However, hepatocytes show modest increase in apoptosis but major increase in necrosis in septic animals [35]. This indicates that the mode of cell death and damage pattern is tissue-specific [10]. Sepsis patients showed increased levels of both apoptotic and necrotic cell death markers [36]. However, in severe septic patients with hepatic dysfunction, the loss of parenchymal cells due to increased necrosis was found to be the primary mode of cell death [36]. This suggested that, unlike apoptosis, hepatocyte necrosis is an early predictor of disease outcome in septic patients with liver dysfunction. On the other hand, it has also been reported that patients who died about six days after the onset of sepsis do not have much tissue necrosis observed in heart and kidneys [37]. With the accumulated studies, it is still not clear whether necrosis or apoptosis plays the predominant role in contributing to the severity of sepsis.

In agreement with the earlier report [24], we demonstrate that, even in our very severe model of CLP-induced sepsis, RIPK3 deficiency delayed mortality. Duprez *et al*. had shown that in their CLP-model, after severe-CLP the WT mice showed 70% mortality on day 3, whereas the majority of *Ripk3*^*-/-*^ mice (>80%) survived. However, unlike the previous study, our CLP-induced sepsis model shows much more severity with 100% mortality on day 2 after CLP in the WT mice group, and the *Ripk3*^*-/-*^ mice group shows delayed mortality but still 100% mortality on day 6 after CLP. Our sepsis model is more representative of the refractory shock and massive systemic inflammation phase that leads to acute multiorgan failure with associated tissue necrosis, which represents a sub-fraction of sepsis cases in clinic. Whether RIPK3 plays a role in regulating the immunosuppressive phase of sepsis and altering the susceptibility to secondary infections needs to be further addressed in a milder CLP model and/or a second hit model with bacterial infection. Although, under very severe conditions, eliminating RIPK3 cannot provide a beneficial effect on the long-term survival, its effect on prolongation of survival time suggests that inhibition of necroptosis may extend the treatment window for other therapeutic agents.

The previous study on polymicrobial sepsis in the absence of RIPK3 has only shown reduced systemic DAMPS, cytokines and LDH but did not investigate the organ specific damage [24]. The lung is the first and most seriously injured organ during sepsis-induced multiple organ failure. Indeed, we show a decrease in the lung histological injury score and the number of apoptotic cells, indicative of reduced ALI in RIPK3-deficient mice. We also find decreased local IL-6 production and neutrophil-attracting chemokine expression in the lungs lacking RIPK3. As migration of neutrophils into the lungs plays a key role in inducing ALI, we show that the dramatic neutrophil infiltration in the lungs seen after CLP in control mice reduces by almost half when RIPK3 is depleted. Interestingly, we noted that the decrease in the numbers of other lung lymphocytes (CD4, CD8, NK and natural killer T cells (NKT)) after CLP was not affected by RIPK3 presence or absence (data not shown). We also note no change in the sepsis-induced decrease of various lymphocyte subsets in the thymus and spleen of *Ripk3*^*-/-*^ mice in comparison with WT mice (data not shown), which suggests that the lymphocyte loss due to apoptosis does not depend on RIPK3-driven necroptosis in sepsis. Thus, the trafficking of only neutrophils, not other immune cells, was affected in the lungs by RIPK3 deficiency in septic mice.

Considering that necrosis plays a major role in sepsis induced liver dysfunction, we next wanted to look at the effect of RIPK3 deficiency in sepsis-induced liver damage. As we expected, the ALT levels (used as liver damage indicator) which increased in WT mice after CLP are significantly reduced by 31.5% in the absence of RIPK3. We show that the chemokine expression in liver tissue is also severely decreased in the absence of RIPK3. Next, we demonstrate that similar to the lungs, the dramatic neutrophil infiltration in the liver seen after CLP in control mice is also reduced by almost half when RIPK3 is depleted. This suggests reduced chemotactic infiltration of neutrophils in RIPK3-deficient livers. Interestingly, we note that RIPK3-deficient mice still show liver damage after CLP-induced sepsis in our model, though much less than WT mice. This also points toward the fact that eliminating necroptosis is beneficial but it is not the only factor contributing to liver damage during severe sepsis, leaving a role for apoptosis and passive necrosis.

NK cells have been shown to participate in the early eradication of bacteria in the liver, peritoneal lavage and circulation during sepsis, likely due to their interactions with macrophages [38]. However, excessive migration of NK cells into the peritoneal cavity amplifies the proinflammatory activities of myeloid cells during septic shock [29]. In this study we report for the first time an increase in the hepatic NK cell population after CLP-induced sepsis specifically in the liver (not seen in lungs) which is attenuated after RIPK3 deficiency. We also noted that 20 hours after CLP, the intensity and level of increased CD69 expression on liver NK cells is similar between *Ripk3*^*-/-*^ mice and WT mice (data not shown), which suggests that RIPK3 deficiency does not affect the activation of NK cells in sepsis, but rather decreases their infiltration in the liver. This is in agreement with the previous study which showed no difference in the capacity of the *Ripk3*^*-/-*^ mice to clear infection in their CLP-induced sepsis model [24]. When monitoring the immune cell trafficking, we did not treat the septic mice with antibiotics in order to obtain the maximal deterioration after CLP. However, in the survival study, septic mice were immediately administered antibiotics after CLP. Whether lowering the bacterial load by antibiotics can change the pattern of immune cell trafficking in RIPK3 deficient mice after CLP needs further investigation.

Sherwood *et al.* have shown that double deficiency of NK cells and CD8T cells is beneficial for survival in sepsis [30,31]. As reported previously [32], we find that hepatic CD8T cells are significantly increased after CLP as compared to sham in the WT mice but not in *Ripk3*^*-/-*^ mice. Interestingly, our study shows that RIPK3 deficiency results in a liver specific combined decrease in NK and CD8T cells after sepsis. The reason or mechanism for accumulation of CD8^+^ T cells as well as NK cells in the septic liver is not yet clear. The possibility of a detrimental role for CD8^+^T cells in liver injury, particularly via activation of local tissue inflammation and apoptotic signaling in sepsis has been suggested [32]. Whether hepatic NK cells have a direct role in causing liver injury and inflammation during sepsis needs to be further investigated. However, cytotoxicity of hepatic NK cells in the liver has been reported previously [39,40], although not in sepsis. Since we observe almost no increase in liver-specific NK and CD8T cells after sepsis in the absence of RIPK3, it is reasonable to suggest that recruitment of these cell subsets to the liver is possibly associated with necrotopic liver damage.

## Conclusions

In conclusion, data provided in this study show that RIPK3 plays a role in altering sepsis-induced immune cell trafficking in an organ-specific manner and its deficiency attenuates organ damage as well as modestly prolongs survival.

### Key messages

• RIPK3 deficiency prolongs survival in severe septic mice induced by CLP and reduces systemic levels of pro-inflammatory cytokines as well as organ injury markers.

• RIPK3 deficiency attenuates CLP-induced ALI resulting in improved integrity of lung architecture, reduced apoptotic cell death in lung epithelium and decreased local pro-inflammatory cytokine and chemokine production.

• RIPK3 deficiency reduces CLP-induced neutrophil infiltration in the lungs, whereas in the liver it not only decreases the neutrophil infiltration but also decreases NK and CD8T cell accumulation.

• RIPK3 deficiency attenuates CLP-induced organ injury by inhibiting the infiltration of immune cells in an organ-specific manner and, thus, protects against sepsis.

## Abbreviations

Abs: antibodies; ALI: acute lung injury; ALT: alanine aminotransferase; AST: aspartate aminotransferase; CLP: cecal ligation and puncture; DAMPs: damage-associated molecular patterns; ELISA: enzyme-linked immunosorbent assay; H & E: hematoxylin and eosin; IL: interleukin; KC: keratinocyte-derived chemokine; LDH: lactate dehydrogenase; MIP: macrophage inflammatory protein; MPO: myleoperoxidase; NK: natural killer; PBS: phosphate-buffered saline; RIPK3: receptor-interacting protein kinase 3; *Ripk3*^*-/-*^: RIPK3-deficient mice; TNF-α: tumor necrosis factor alpha; WT: wild type.

## Competing interests

The authors declare that they have no competing interests.

## Authors’ contributions

AS and SM carried out all animal experiments, performed biochemical measurements, analyzed the data and conducted the statistical analysis. AS drafted the manuscript. WLY initiated the project, designed the experiments, assisted in data analysis and interpretation, and critically revised the manuscript. ZW performed animal experiments. PW conceived the study, participated in analysis and interpretation of data, and critically reviewed and approved the manuscript. All authors read and approved the final manuscript.
